# Fluoxetine increases astrocytic glucose uptake and glycolysis in corticosterone-induced depression through restricting GR-TXNIP-GLUT1 Pathway

**DOI:** 10.3389/fphar.2022.872375

**Published:** 2022-08-29

**Authors:** Shu-Man Pan, Yi-Fan Zhou, Na Zuo, Rui-Qing Jiao, Ling-Dong Kong, Ying Pan

**Affiliations:** ^1^ State Key Laboratory of Pharmaceutical Biotechnology, School of Life Sciences, Nanjing University, Nanjing, Jiangsu, China; ^2^ State Key Laboratory of Natural Medicines, China Pharmaceutical University, Nanjing, Jiangsu, China

**Keywords:** fluoxetine, astrocyte, glycolysis, GR, TXNIP-GLUT1 pathway

## Abstract

Antidepressant fluoxetine can affect cerebral glucose metabolism in clinic, but the underlying molecular mechanism remains poorly understood. Here, we examined the effect of fluoxetine on brain regional glucose metabolism in a rat model of depression induced by repeated corticosterone injection, and explored the molecular mechanism. Fluoxetine was found to recover the decrease of ^18^F-fluorodeoxyglucose (^18^F-FDG) signal in prefrontal cortex (PFC), and increased 2-[*N*-(7-Nitrobenz-2-oxa-1,3-diazol-4-yl) amino]-2-deoxy-D-glucose (2-NBDG, a fluorescent glucose analog) uptake in an astrocyte-specific manner in *ex vivo* cultured PFC slices from corticosterone-induced depressive rats, which were consistent with its improvement of animal depressive behaviors. Furthermore, fluoxetine restricted nuclear translocation of glucocorticoid receptor (GR) to suppress the transcription of *thioredoxin interacting protein* (*TXNIP*). Subsequently, it promoted glucose transporter 1 (GLUT1)-mediated glucose uptake and glycolysis of PFC astrocytes through suppressing TXNIP expression under corticosterone-induced depressive state. More importantly, fluoxetine could improve glucose metabolism of corticosterone-stimulated astrocytes via TXNIP-GLUT1 pathway. These results demonstrated that fluoxetine increased astrocytic glucose uptake and glycolysis in corticosterone-induced depression via restricting GR-TXNIP-GLUT1 pathway. The modulation of astrocytic glucose metabolism by fluoxetine was suggested as a novel mechanism of its antidepressant action.

## 1 Introduction

Depression is a common mental disease, causing severe psychological and physical disorders, even suicide (Malhi and Mann, 2018). Fluoxetine is a Food and Drug Administration approved drug as first-line therapy for depression. Although developed as a selective inhibitor of serotonin reuptake, fluoxetine is reported to exert neuroprotection (Hui et al., 2014; Levy et al., 2019) and anti-inflammation activity (Garcia-Garcia et al., 2022), possibly contributing to its antidepressant effect independent of serotonin uptake. Of note, the improvement of brain glucose metabolism by fluoxetine is observed in patients with major depression (Zhang et al., 2006). More importantly, the response to fluoxetine treatment is associated with regional glucose metabolism rate of depressive patients (Mayberg et al., 2000). However, the molecular mechanism by which fluoxetine influences brain glucose metabolism in depressive condition is still unknown.

Intracerebral glucose is mainly uptake by astrocytes *via* glucose transporter 1 (GLUT1) and metabolized to lactate (Dienel, 2019). In cultured brain slices, astrocytes uptake more glucose analogue (NBDGs) than neurons (Barros et al., 2009; Jakoby et al., 2014). Whisker stimulation rapidly accelerates glucose uptake of astrocytes but not neurons in the barrel cortex of rats (Chuquet et al., 2010). Astrocyte-derived lactate supports synaptic plasticity (Murphy-Royal et al., 2020) and promotes neuronal survival (Muraleedharan et al., 2020). Fluoxetine is reported to stimulate glucose utilization and lactate release in normal primary cortical astrocytes (Allaman et al., 2011). Whereas, the influence of fluoxetine on astrocytic glucose metabolism in depressive condition is unknown.

Thioredoxin-interacting protein (TXNIP) as an endogenous inhibitor of the thioredoxin (TRX) system regulates redox stress and inflammation. Brain TXNIP is increased in stroke, Alzheimer’s disease and other central nervous system disease (Nasoohi et al., 2018). Over-expression of TXNIP *via* plasmid construct transfection stimulates GLUT1 internalization in TRVb-1 cells (Wu et al., 2013). Recently, we found that high TXNIP level restricted GLUT1-mediated glucose uptake and caused glucose hypometabolism of astrocytes in corticosterone-induced depressive state (Pan et al., 2021). However, the effect of fluoxetine on astrocytic TXNIP has not been reported. The question arose whether fluoxetine improves astrocytic glucose metabolism through TXNIP-GLUT1-mediated glucose uptake in corticosterone-induced depression. Hyper-activation of the hypothalamic-pituitary-adrenal (HPA) axis with high cortisol or corticosterone level is observed in depressive patients or animals (Menke, 2019). The ligand-bound GR complex translocate into the nucleus and bind to glucocorticoid response elements in promoter or intragenic region of target genes to regulate the transcription of various genes, such as *TXNIP* (Kadmiel and Cidlowski, 2013). There are functional glucocorticoid response elements in murine *TXNIP* promoter (Wang et al., 2006). Dexamethasone (a kind of synthetic glucocorticoid) is reported to induce TXNIP over-expression in both human and murine T cells (Wang et al., 2006; Stolearenco et al., 2021). But the relationship between glucocorticoid signaling pathway and TXNIP level in astrocytes is still unknown.

**FIGURE 1:**
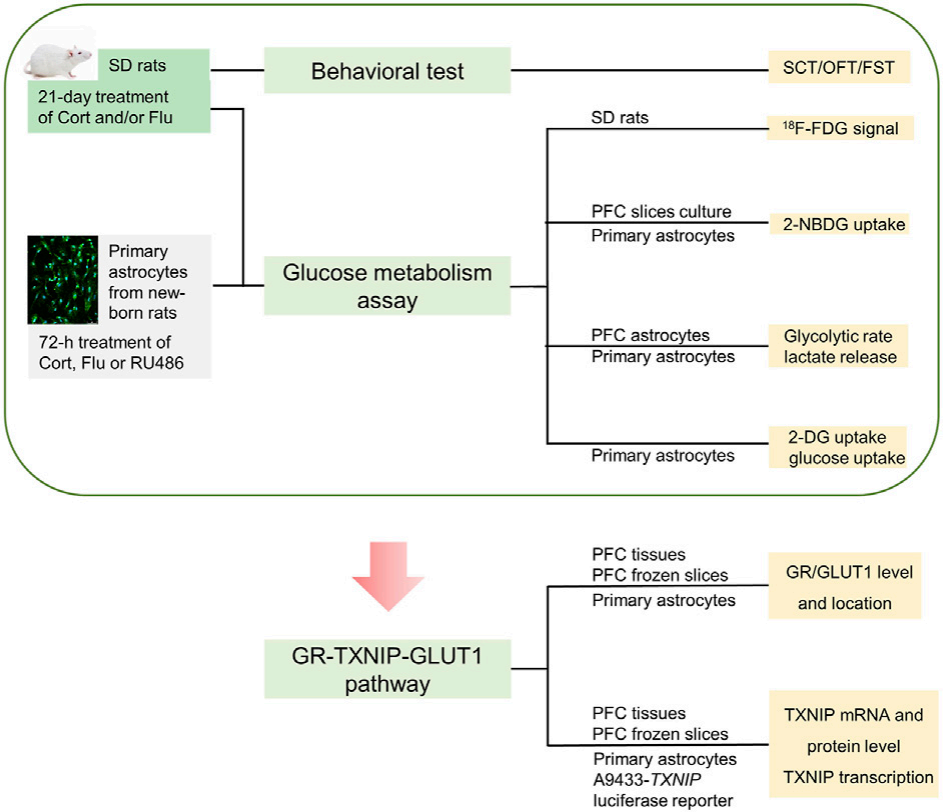
Schematic of this study.

Thus, we hypothesized fluoxetine might modulate brain astrocytic glucose metabolism to improve cerebral glucose metabolism in depressive condition. We aimed to illustrate the influence of fluoxetine on astrocytic glucose metabolism in corticosterone-induced depressive condition and explore the underlying mechanism. Firstly, ^18^F-FDG positron emission tomography (PET) was applied to analyze the effect of fluoxetine on glucose metabolism in brain regions of corticosterone-induced depressive rats. 2-NBDG uptake, 2-DG uptake, glucose uptake, glycolysis rate and supernatant lactate level were detected to examine the action of fluoxetine on astrocytic glucose metabolism in corticosterone-induced depression, Furthermore, we tried to explore whether GR-TXNIP-GLUT1 pathway play an important role in astrocytic glucose metabolism disturbance in corticosterone-induced depression, and more importantly, to investigate the influence of fluoxetine on this pathway. This study creatively illustrated that fluoxetine might improve cerebral glucose metabolism through modulating astrocytic glucose metabolism and provided new mechanism for its antidepressant activity. Process of this study was shown in Figure 1.

## 2 Materials and methods

### 2.1 Animals

To avoid the behavioral influence of estrogen, male rats were selected as experimental animals (Albert and Newhouse, 2019). Sprague-Dawley rats (male, 6–8 weeks old) were purchased from Vital River Laboratory Animal Technology Co., Ltd. (Beijing, China) and housed in specific pathogen free facilities with controlled temperature (23 ± 2°C) and humidity (50% ± 5%). Rats were given food and water *ad libitum* in a 12 h light (9:00–21:00)/dark cycle.

After 1-week adaption to environment, animals were assigned to different groups arbitrarily. According to previous studies (Eydipour et al., 2020) and our preliminary study, 21-day continuous corticosterone treatment (40 mg/kg/day) was used to induce animal model of depression. Corticosterone was suspended in saline solution supplied with 1‰ DMSO and 1‰ Tween 80 and given to rats through subcutaneous injection. Rats in control group received the same amount of saline solution (with 1‰ DMSO and 1‰ Tween 80) each day. To study the efficacy and mechanism of fluoxetine, fluoxetine hydrochloride (10 mg/kg/day, with saline as vehicle) was given to corticosterone-stimulated rats by gavage for 21 day. The dosage of fluoxetine hydrochloride (10 mg/kg) was chosen referred to clinical medication guidelines (Avasthi and Grover, 2018) and our previous studies (Pan et al., 2014; Jia et al., 2018).

### 2.2 Behavioral tests

After 21-day treatment of corticosterone or/and fluoxetine, behavioral tests were conducted within 10:00–15:00 as shown in Figure 1.

#### 2.2.1 Sucrose consumption test

Sucrose consumption test (SCT) was conducted to evaluate anhedonia, a core symptom of depression (Willner et al., 1987). The procedure of SCT was described in our previous study (Jia et al., 2018; Pan et al., 2021). Generally, rats were trained to consume 1% sucrose solution (w/v) before test. After adaption procedure, sucrose solution that consumed during 1 h (12:00–13:00) was measured and normalized by body weight of fasting animal (Sucrose consumption = Sucrose consumption in 1 h/Body weight of animal).

#### 2.2.2 Open field test

Open field test (OFT) was applied to evaluate the locomotor activity and anxiety behavior of rats. The area of experimental apparatus (0.5 m × 0.5 m × 0.6 m) was divided into nine equal squares, one central zone and eight peripheral zones. Rat was placed to the centrum of the field. Then, behaviors of rats in the field were recorded for 10 min. The movements and position of rats during 10 min observation period were analyzed by Supermaze software (Xinruan Information Technology Co. Ltd., Shanghai, China).

#### 2.2.3 Forced swimming test

Forced swimming test (FST) is frequently used to evaluate the antidepressant activity of compounds in animal models. FST was conducted as described (Slattery and Cryan, 2012). Briefly, the glass cylinder was filled with 23–25°C water to a depth of 30 cm. The cylinder was 20 cm in diameter. Rat was put into the cylinder for pre-swim. After 15 min of pre-swim, rat was removed from water, dried with towels and put into warm cage. 24 h after pre-swim, glass cylinder was refilled with 23–25°C water. Rat was put into water to swim for 6 min. The behavior of animal was continuously recorded by camera. Behaviors of animal were divided into three different status (immobile, swimming, struggle). The definition of status refers to our previous work (Pan et al., 2021). Time of different status in FST was calculated by SuperFst software (Xinruan Information Technology Co. Ltd., Shanghai, China).

### 2.3 ^18^F-fluorodeoxyglucose positron emission tomography scan

Regional brain glucose metabolism was detected by ^18^F-FDG PET/CT scans. Animals (*n* = 4 each group) were randomly selected for this experiment. This work was conducted with the help of MITRO Biotec (Nanjing, China). Concisely, following 12 h fasting, isoflurane (1.5–2.5%, 300–800 ml/min) was applied to anaesthetize rats. Then, 300 μCi ^18^F-FDG was administrated to rats via tail vein injection. 1 h post injection of ^18^F-FDG, static images were acquired for 10 min using the small animal PET/CT (Inveon, Siemens). A two bed CT scan (80 kV, 500 mA) was obtained for attenuation corrections of PET images. PET images were acquired in a list mode with a coincidence timing window of 1.5 ns. All PET images were reconstructed using OSEM3D/MAP (2 OSEM3D iterations, 16 MAP iterations). PET image dimensions were 128 voxels × 128 voxels × 159 voxels with a voxel size of 0.7764 mm × 0.7764 mm × 0.796 mm (Pan et al., 2021). PMOD software (version3.805, PMOD Technology, Switzerland) was used to analyze PET images. The SUV (standardized uptake value) of different brain regions was obtained.

### 2.4 2-NBDG uptake by SR101 + cells in prefrontal cortex slices

After 21 day treatment of corticosterone and fluoxetine, prefrontal cortex (PFC) slices were prepared and cultured in oxygen saturated artificial cerebrospinal fluid according to previous report (McNair et al., 2017). 2-[*N*-(7-Nitrobenz-2-oxa-1,3-diazol-4-yl) amino]-2-deoxy-D-glucose (2-NBDG, a fluorescent glucose analog) can be taken up by cells through glucose transporters just like glucose. Therefore, 2-NBDG (5 μM) was added into artificial cerebrospinal fluid to evaluate glucose metabolism of PFC slices for 1 h. Sulforhodamine 101 (SR101) was used as a marker of astrocytes because they uptake it selectively (Nimmerjahn et al., 2004; Kafitz et al., 2008). PFC slices were cultured in artificial cerebrospinal fluid with SR101 (0.5 μM) for 20 min. 2-NBDG signal of SR101 + cell in PFC slice was observed by two-photon confocal microscopy. The cell region of astrocytes was manually framed according to SR101 signal. Then, fluorescence intensity of 2-NBDG in this cell region was measured by ImageJ.

### 2.5 Glucose transporter 1 translocation detection by flow cytometry

After behavioral tests, rats were decapitated. PFCs were quickly separated, cut into pieces, digested by papain with 0.1% DNase. Cell debris was removed and all cells were enriched by Percoll (17-0891-02, GE Healthcare). The isolated cells were fixed by 1% PFA. The fixed cells were incubated with primary antibodies rabbit anti-GLAST1 (1:100, ab416, Abcam) and mouse anti-GLUT1 (1:100, ab40084, Abcam) in permeabilized (0.1% Triton, for total GLUT1 detection) or non-permeabilized (without Triton, for plasma membrane GLUT1 detection) condition for 1 h. Then, cells were incubated with secondary antibody Alexa Flour 488 goat anti-rabbit IgG and Alexa Flour 647 goat anti-mouse IgG for 0.5 h. The fluorescence signal was detected by flow cytometer (Attune NxT, Invitrogen). Astrocytes were identified by GLAST1 expression (Norden et al., 2016).

### 2.6 Culture and treatment of primary astrocyte

#### 2.6.1 Isolation and culture of primary astrocytes

PFC was extracted from 1 to 2 day postnatal Sprague-Dawley male rat and separated into single cell by 0.25% trypsin. DMEM/F12 medium supplemented with 10% fetal bovine serum (FBS) and 1% penicillin-streptomycin was used to culture cells. 24 h after seeding cells, cultured medium was changed to remove debris. When cell confluence reached about 80%, flasks were shaken at a speed of 200 r/min in 37°C to remove microglia and oligodendrocytes (Schildge et al., 2013).

#### 2.6.2 Treatment of primary astrocytes

Primary astrocytes were plated in culture plate. Hundred Nanometre corticosterone was chosen to stimulate primary astrocytes according to previous study (Murphy-Royal et al., 2020; Pan et al., 2021). After evaluating the dosage effect of fluoxetine on astrocytic lactate release and cell activity (Supplementary Figure S1), 5 μM fluoxetine was used to stimulate *in vitro* cultured primary astrocytes directly. It is worth mentioning that corticosterone (100 nM) and fluoxetine (5 μM) had no obvious effect on cell activity and biomass (Supplementary Figure S2). RU486 (a GR antagonist, 1 μM) was used to inhibit the activity of GR. Verapamil, a reported *TXNIP* transcription inhibitor (Xu et al., 2012; Harper et al., 2019), was used as positive control in *TXNIP* transcription-related experiments. Different indicators were detected after 72 h treatment of corticosterone, fluoxetine or RU486.

### 2.7 Glucose uptake of primary astrocytes

Three different methods were used to evaluate glucose uptake activity of primary astrocytes.

#### 2.7.1 2-NBDG uptake by primary astrocytes

Primary astrocytes were seeded in 96-well plate at a density of 3,000 cells/well. After 72 h treatment of corticosterone and fluoxetine, 2-NBDG uptake ability of astrocytes was evaluated. 2-NBDG was dissolved in phosphate-buffered saline (PBS) to get staining solution. Cultured medium was removed and cell was rinsed by PBS. Then, primary astrocytes were incubated with 50 μM 2-NBDG at 37°C for 30 min. After incubation, staining solution was removed and cell was washed by hank’s balanced salt solution (HBSS). Fluorescence intensity of 2-NBDG (Ex/Em = 465/540 nm) was detected by microplate reader.

#### 2.7.2 Direct glucose uptake assay

Hexokinase is the first enzyme that metabolize glucose. We could detect glucose that has been taken into cells directly *via* inhibiting hexokinase activity. Generally, primary astrocytes were seeded at a density of 40,000 cells/well in 6-well culture plate. After treatment of corticosterone or/and fluoxetine for 72 h, culture medium (DMEM/F12 medium supplemented with 10% FBS) was removed and cell was washed by PBS at 37°C. Then, primary astrocytes were incubated with glucose- and FBS-free medium for 30 min of starvation. Next, primary astrocytes were incubated with medium containing 10 mM glucose and 10% FBS for glucose uptake. Notably, hexokinase inhibitor was added into medium to inhibit the activity of hexokinase and prevent the metabolism of glucose. After glucose uptake period, iced-cold PBS supplemented with hexokinase inhibitor was used to wash cells. Then, primary astrocytes were lysed by lysis buffer with hexokinase inhibitors. The glucose in cell lysate was oxidized by enzyme reactions and converted to red probe with fluorescence (ab234043, Abcam). The level of end product was measured by microplate reader (Ex/Em = 535/587 nm). Protein level of cell lysate was detected by BCA assay kit (23250, Thermo scientific) to normalize result.

#### 2.7.3 2-deoxyglucose uptake assay

2-deoxyglucose (2-DG) is the analogue of glucose. It could be transported into cell and rapidly phosphorylated in the same way as glucose. Differently, 2-DG6P, the phosphorylated product of 2-DG, cannot be metabolized further. Therefore, intracellular accumulated 2-DG6P was detected to evaluate glucose uptake ability of astrocytes. Firstly, after treatment of corticosterone and fluoxetine, astrocytes were starved by glucose- and FBS-free medium for 30 min. Then, astrocytes were incubated with medium that supplied with 1 mM 2-DG for 30 min. After 2-DG uptake, an acid detergent solution was added to terminate 2-DG uptake. Next, high-pH buffer solution was used to neutralize the sample. 2-DG6P in sample was oxidized by G6PDH. G6PDH catalyzed end product could be detected *via* bioluminescent reaction (J1342, Promega). Importantly, before adding high-pH buffer solution, 20 μl solution was taken out from each sample for protein concentration detection. Intracellular 2-DG6P level was normalized by protein level.

### 2.8 Seahorse glycolytic rate assay

Primary astrocytes were plated at a density of 2,000 cells/well in XF96 plate. The procedures of the glycolytic rate assay were conducted according to the user guide of Agilent Seahorse XF Glycolytic Rate Assay Kit (103344-100, Agilent Technologies). The day before assay, assay medium was prepared by supplementing base medium with 10 mM glucose, 1 mM pyruvate, 2 mM glutamine and 5.0 mM HEPES (pH 7.4). After 72 h stimulation with corticosterone and fluoxetine, cells were washed with warm assay medium for two times and the XF96 cell culture plate was calibrated in a non-CO_2_ incubator at 37°C for 1 h. After calibration, OCR (oxygen consumption rate) and ECAR (extracellular acidification rate) were measured by seahorse analyzer. Rotenone/antimycin A (0.5 μM) and 2-DG (5 mM) were added to detect the compensatory glycolysis and post 2-DG acidification. GlycoPER (glycolytic proton efflux rate) was calculated according to the values of OCR and ECAR. After seahorse glycolytic rate assay, the DNA level per cell was detected by commercial kit (C7026, Invitrogen). The results of glycoPER were normalized by cell content (the ratio of DNA level).

### 2.9 Supernatant lactate measurement

Supernatant lactate level was measured by L-lactate assay kit (ab65330, Abcam). All the operations followed the manufacture’s instruction. The protein contents of primary astrocytes were detected. All the results were normalized by protein ratio.

### 2.10 Development of a stable thioredoxin-interacting protein -promoter-driven luciferase reporter cell line

A human *TXNIP* promoter (> 1518bp + 50 UTR, Supplementary Table S1) connected with luciferase reporter was cloned to a plasmid. Then, plasmid containing *TXNIP* promoter, helper plasmids (pGag/Pol, pRev, pVSV-G) and RNAi-mate were added to the medium of HEK293T cells for lentivirus production. Subsequently, *TXNIP*-luc expressing virus was transduced to U87MG cells. Through puromycin selection, a polyclone of U87MG cells with stable expression of *TXNIP*-luc was obtained. The stable *TXNIP*-promote**-**driven luciferase reporter cell line was developed by Gene Pharm (Shanghai, China). We named the stable cell line “A9433.” A9433 cell was maintained in DMEM medium in the presence of 1 μg/ml puromycin. The activity of firefly luciferase was detected by commercial kit (RG006, Beyotime).

### 2.11 Western blot

Nuclear, cytoplasmic (CYT) and plasma membrane (PM) protein of PFC tissues or astrocytes were isolated by commercial kits (KGP1100, KeyGEN BioTECH; P0033, Beyotime Biotechnology). Phenylmethanesulfonyl fluoride (PMSF, a protease inhibitor) was used to inhibit protein degradation. The protein concentration of sample was detected by bicinchoninic acid (BCA) protein assay kit (Thermo scientific). Lysates were loaded into 10% sodium dodecyl sulfate-polyacrylamid gel electrophoresis and subsequently transferred to polyvinylidene fluoride membranes. Membranes were blocked in 5% non-fat milk for 1 h. Then, the following first antibodies were used: GLUT1 (1:5000, ab115730, Abcam), TXNIP (1:1000, #14715, CST), Na, K ATPase (1:1000, #3010, CST), GR (1:1000, #3600, CST), Histone H3 (1:500, sc-517576, Santa Cruz) and β-actin (1:2000, #4970, CST). After incubating with first antibody overnight, membranes were incubated goat anti-mouse IgG (1:5000, HAF007, R&D systems) or goat anti-rabbit IgG (1:3000, #7074, CST) for 1.5 h. Protein bands were visualized by ECL (#180–5001, Tanon) and imaged by chemiluminescence imaging instrument (#5200, Tanon). The gray value of images was measured by ImageJ.

### 2.12 Immunofluorescence

#### 2.12.1 Detection prefrontal cortex astrocytic glucocorticoid receptor and thioredoxin-interacting protein level by immunofluorescence

After behavioral tests, the brains of rats with corticosterone or/and fluoxetine treatment were separated after perfusion of 4% paraformaldehyde (PFA). Subsequently, brains were post-fixed by 4% PFA and dehydrated by 10%, 20% and 30% sucrose solution (w/v). Then, brains were embedded by optimal cutting temperature compound after trimming and cut into sections by cryostat (CM 1950; Leica). The frozen sections were stored at −80°C before immunofluorescence staining. After recovering to room temperature and PBS washing, the frozen sections were incubated with GFAP (1:200, #13-0300, Thermo scientific), TXNIP (1:50, sc-271238, Santa Cruz) or GR (1:200, #3600, CST) at 37°C for 4 h. Then, secondary antibodies were added. DAPI (C1002, Beyotime Biotechnology) was used to stain nucleus. To avoid fading of fluorescence, samples were mounted by anti-fade mounting medium (P0126, Beyotime Biotechnology). Fluorescence images were obtained by two-photon confocal microscope (TCS SP8-MaiTai M, Leica) and analyzed by Image J.

#### 2.12.2 Detection glucocorticoid receptor translocation of astrocytes by immunofluorescence

After corticosterone and fluoxetine treatment, primary astrocytes were fixed by 4% PFA. Then, astrocytes were incubated with GR (1:200, #3600, CST) overnight and secondary antibody Alexa Flour 488 goat anti-rabbit IgG for 0.5 h. Nucleus of primary astrocytes were labeled by DAPI (C1002, Beyotime Biotechnology). The images of GR were randomly captured by Harmony system (Operetta) and analyzed by Columbus system. For image analysis, firstly, cytoplasmic (CYT) and nuclear regions of astrocytes were identified in Columbus system. Then, GR level in nuclear or CYT region was calculated.

### 2.13 qRT-PCR analysis

Total RNA of PFC tissues or primary astrocytes was extracted by Trizol (15596018, Invitrogen). Isolated RNA was dissolved in diethyl pyrocarbonate (DEPC) H_2_O. HiScriptⅡqRT Supermix (Vazyme) was used to reverse RNA to cDNA. Expression of *TXNIP* was quantified using qRT-PCR. The primers used in this study were listed as follows: *TXNIP*, sense, TTTCTGCCTCTCTGCTTG and antisense, GAC​TTG​CCT​ACT​GAT​TGC​C; *β-Tubulin*, sense, CATCACAGGCAAGGAAGA and antisense, GTG​GAA​AAC​CAA​GAA​GCC. SYBR^®^ Green was combined with DNA during amplification (Bio-Rad CFX96). Relative expression of *TXNIP* was analyzed by 2^−△△Ct^ method with *β-Tubulin* as internal control.

### 2.14 Statistical analysis

Data were expressed as mean ± SEM and statistical analyzed by Prism 7 (GraphPad Software, San Diego, CA, United States). Data with normal distribution were analyzed by one-way ANOVA followed by Dunnett’s post-hoc test for multiple comparisons. Data without normal distribution were analyzed by Kruskal-Wallis test followed by Dunnett’s post-hoc test for multiple comparisons. *p* < 0.05 was regarded as statistically significant.

## 3 Results

### 3.1 Fluoxetine increases prefrontal cortex glucose metabolism

**FIGURE 2:**
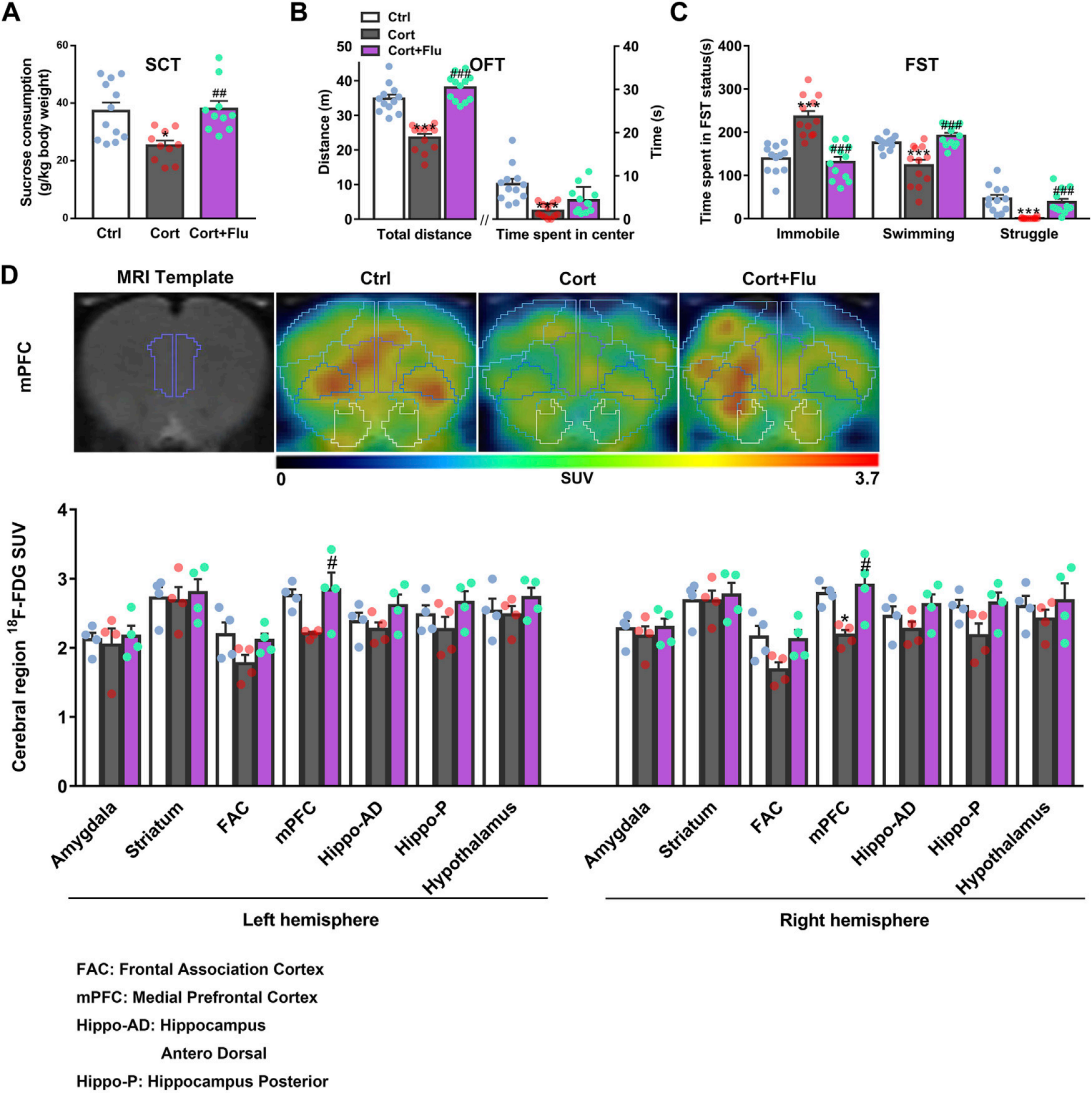
Fluoxetine improves prefrontal cortex glucose metabolism in a rat model of depression induced by repeated corticosterone injection. **(A)** 24 h after the last treatment of fluoxetine (10 mg/kg), behavioral tests were conducted. The sucrose (1%) consumption in SCT, *n* = 10–12 animals/group; *P* CortxCort + Flu = 0.0022. **(B)** Total distance and time spent in center in OFT, *n* = 12 animals/group; total distance: *P* CortxCort + Flu <0.0001; time spent in center: *P* CortxCort + Flu = 0.0765. **(C)** Time spent in different status in FST, *n* = 12 animals/group; Immobile: *P* CortxCort + Flu <0.0001; Swimming: *P* CortxCort + Flu <0.0001; Struggle: *P* CortxCort + Flu = 0.0001. **(D)** Brian ^18^F-FDG PET imaging and quantification of rats, *n* = 4 animals/group; mPFC_l: *P* CortxCort + Flu = 0.0313; mPFC_r: *P* CortxCort + Flu = 0.0102. Note: Frontal association cortex (FAC) and medial prefrontal cortex (mPFC) belong to PFC. All data were expressed as mean ± SEM, ^*^ *p* < 0.05, **p < 0.01, ***p < 0.001 vs. Ctrl (control) group, ^#^ *p* < 0.05, ^##^ *p* < 0.01, ^###^ *p* < 0.001 vs. Cort (corticosterone) group (Normality of data was checked by Shapiro-Wilk test. Data with or without normal distribution were analyzed by one-way ANOVA or Kruskal-Wallis test followed by Dunnett’s post-hoc test for multiple comparisons).

Fluoxetine increased sucrose consumption in SCT (Figure 2A) and locomotor activity in OFT (Figure 2B) as well as reduced despair behavior in FST (Figure 2C) of corticosterone-induced depressive rats. To investigate whether fluoxetine influenced brain glucose metabolism in this rat model of depression, we used ^18^F-FDG PET assay and found that fluoxetine increased SUV of ^18^F-FDG in frontal association cortex (FAC), medial prefrontal cortex (mPFC), hippocampus and hypothalamus in this animal model, showing the enhancement of brain glucose metabolism, especially in mPFC (Figure 2D; Supplementary Table S2). The obvious recovery of corticosterone-induced rat PFC glucose hypometabolism by fluoxetine was consistent with its antidepressant effects.

### 3.2 Fluoxetine increases prefrontal cortex astrocytic glucose uptake and glycolysis

**FIGURE 3:**
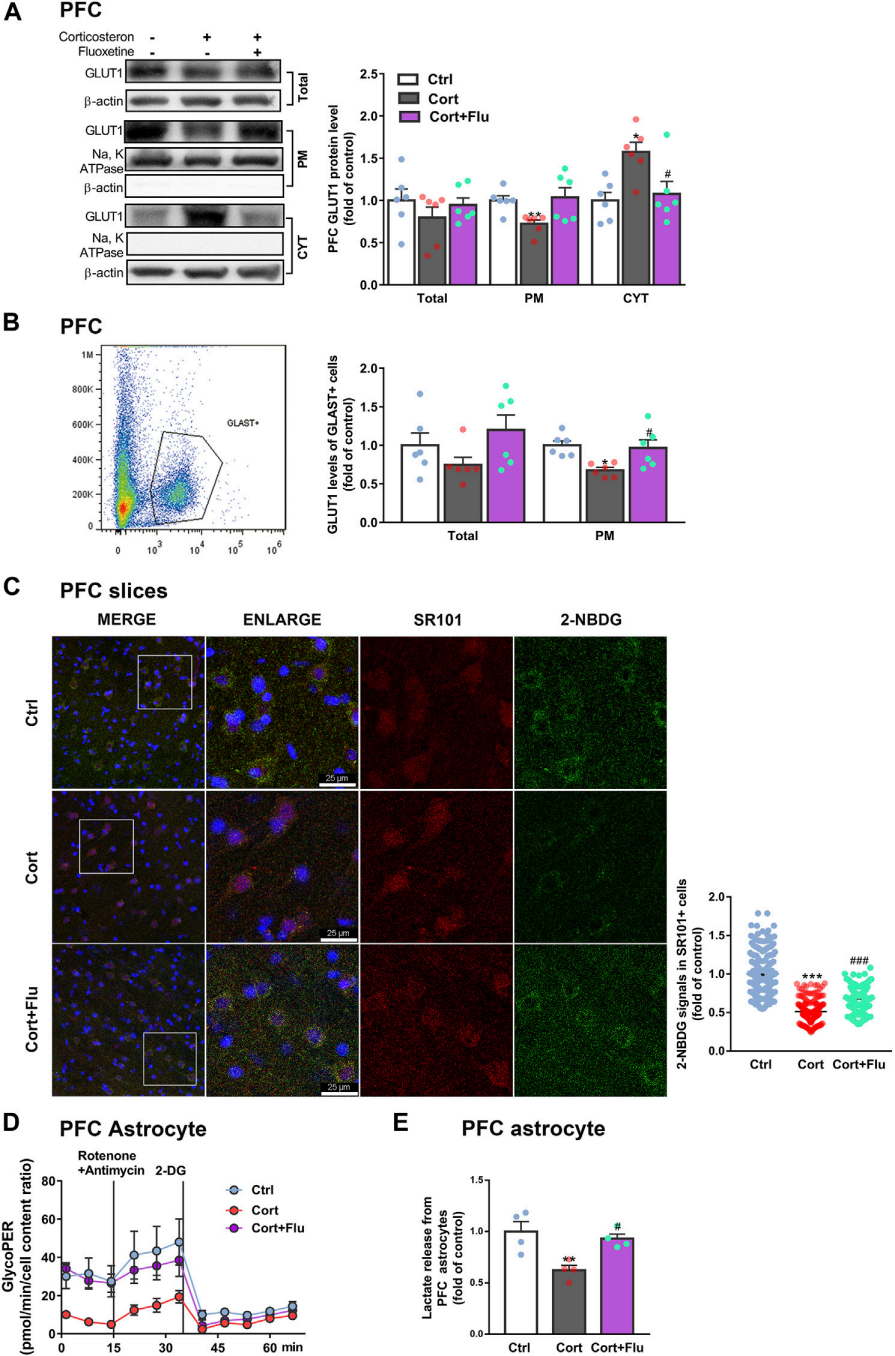
Fluoxetine recovers prefrontal cortex astrocytic glucose uptake and glycolysis in a rat model of depression induced by repeated corticosterone injection. **(A)** GLUT1 (total, PM and CYT fractions) protein levels in PFC of rats treated with fluoxetine (10 mg/kg), *n* = 6 animals/group; Total: *P* CortxCort + Flu >0.9999; PM: *P* CortxCort + Flu = 0.0699; CYT: *P* CortxCort + Flu = 0.0217. **(B)** Total and PM GLUT1 levels in GLAST + astrocytes from PFC of rats treated with fluoxetine (10 mg/kg) were detected by flow cytometry, *n* = 6 animals/group; Total: *P* CortxCort + Flu = 0.1029; PM: *P* CortxCort + Flu = 0.0231. **(C)** The fluorescence intensity of 2-NBDG in SR101 + cells imaged by confocal (scale bar = 25 μm) and analyzed by ImageJ, Ctrl: *n* = 226 cells, Cort: *n* = 195 cells, Cort + Flu: *n* = 205 cells; *P* CortxCort + Flu <0.0001. **(D)** GlycoPER (glycolytic proton efflux) was calculated according to the results of seahorse glycolytic rate assay, *n* = 4 animals/group. **(E)** Lactate released from PFC astrocytes of fluoxetine (10 mg/kg)-treated rats was detected, *n* = 4 animals/group; *P* CortxCort + Flu = 0.0203. All data were expressed as mean ± SEM, *p < 0.05, **p < 0. 01, ***p < 0.001 vs. Ctrl (control) group, ^#^ *p* < 0.05, ^###^ *p* < 0.001 vs. Cort (corticosterone) group (Normality of data was checked by Shapiro-Wilk test. Data with or without normal distribution were analyzed by one-way ANOVA or Kruskal-Wallis test followed by Dunnett’s post-hoc test for multiple comparisons).

Next, fluoxetine was found to increase GLUT1 plasma membrane (PM) translocation in PFC of depressive rats induced by repeated corticosterone injection (Figure 3A). Furthermore, fluoxetine significantly upregulated PM GLUT1 level of GLAST + astrocytes isolated from PFC of this animal model (Figure 3B). Being consistent with the stimulation of GLUT1 PM translocation, fluoxetine increased 2-NBDG uptake of astrocytes (SR101 + cells) in *ex vivo* cultured PFC slices with corticosterone exposure (Figure 3C). Fluoxetine also promoted glycolytic rate (Figure 3D) and enhanced astrocytic lactate release (Figure 3E) in PFC astrocytes isolated from this animal model.

**FIGURE 4:**
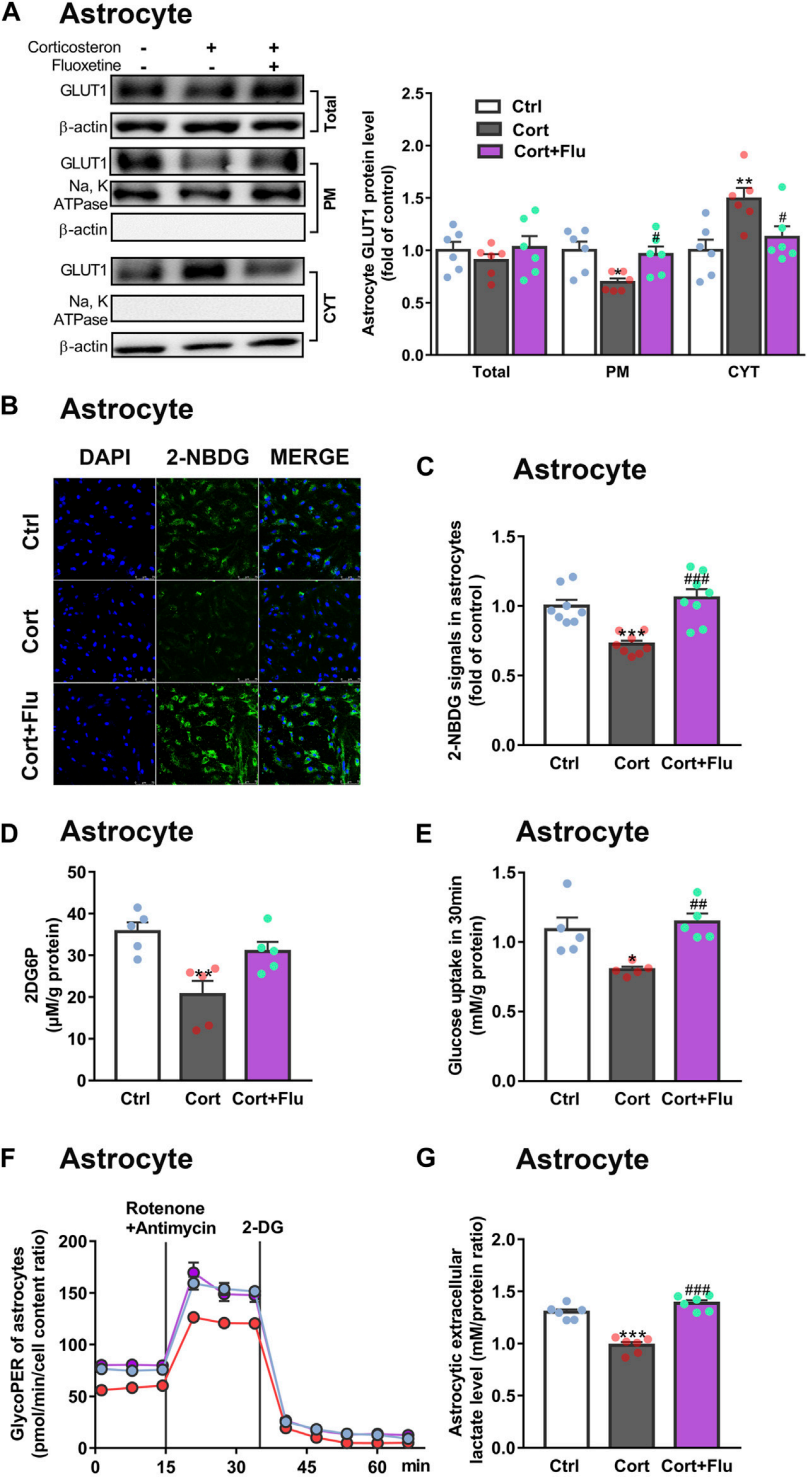
Fluoxetine increases glucose uptake and glycolysis in primary astrocytes cultured with corticosterone *in vitro*. **(A)** GLUT1 (total, PM and CYT fractions) protein levels in fluoxetine (5 μM)-treated primary astrocytes (72 h), *n* = 6 cell cultures/group; Total: *P* CortxCort + Flu = 0.5093; PM: *P* CortxCort + Flu = 0.0287; CYT: *P* CortxCort + Flu = 0.0435. **(B,C)** 2-NBDG uptake in astrocytes treated with fluoxetine (5 μM) were imaged by confocal microscopy [**(B)**, scale bar = 75 μm)] or measured by microplate reader [**(C)**, *n* = 8 cell cultures/group)]; *P* CortxCort + Flu = 0.0001. **(D,E)** 2-DG [**(D)**, *n* = 5 cell cultures/group; *P* CortxCort + Flu = 0.1125)] or Glucose [**(E)**, *n* = 5 cell cultures/group; *P* CortxCort + Flu = 0.0043)] uptake by astrocytes treated with fluoxetine (5 μM) in 30 min **(F,G)** GlycoPER [**(F)**, *n* = 6 cell cultures/group)] and lactate release [**(G)**, *n* = 6 cell cultures/group; *P* CortxCort + Flu <0.0001)] of corticosterone-exposed primary astrocytes with fluoxetine (5 μM) treatment. All data were expressed as mean ± SEM. *p < 0.05, **p < 0.01., ***p < 0.001 vs. Ctrl (control) group, ^#^ *p* < 0.05, ^##^ *p* < 0.01, ^###^ *p* < 0.001 vs. Cort (corticosterone) group (Normality of data was checked by Shapiro-Wilk test. Data with or without normal distribution were analyzed by one-way ANOVA or Kruskal-Wallis test followed by Dunnett’s post-hoc test for multiple comparisons).

Subsequently, fluoxetine-treated primary astrocytes isolated from newborn rats were directly used to study the effect on astrocytic glucose metabolism under stress-level corticosterone condition *in vitro*. Fluoxetine enhanced primary astrocyte PM GLUT1 translocation (Figure 4A), 2-NBDG uptake (Figures 4B,C **)**, 2DG6P uptake (Figure 4D), glucose uptake (Figure 4E), glycolytic rate (Figure 4F) and lactate release (Figure 4G) compared with corticosterone-treated primary astrocytes. In Figures 3D,E, primary astrocytes were isolated from PFC of depressive rats induced by repeated corticosterone injection after behavioral tests. In Figure 4, primary astrocytes were isolated from PFC of newborn rat and treated with corticosterone *in vitro*. Because of the difference of cell age and cell density, the glycoPER vale in Figures 3D, 4F was different. But, both the *in vivo* (Figure 3D) and *in vitro* (Figure 4F) data showed that fluoxetine increased astrocytic glycolysis. These data indicated that fluoxetine increased astrocytic PM GLUT1 translocation, and subsequent glucose uptake and glycolysis, resulting in recovery of astrocytic glucose hypometabolism in corticosterone-induced depressive condition.

### 3.3 Fluoxetine suppresses astrocytic thioredoxin-interacting protein expression

**FIGURE 5:**
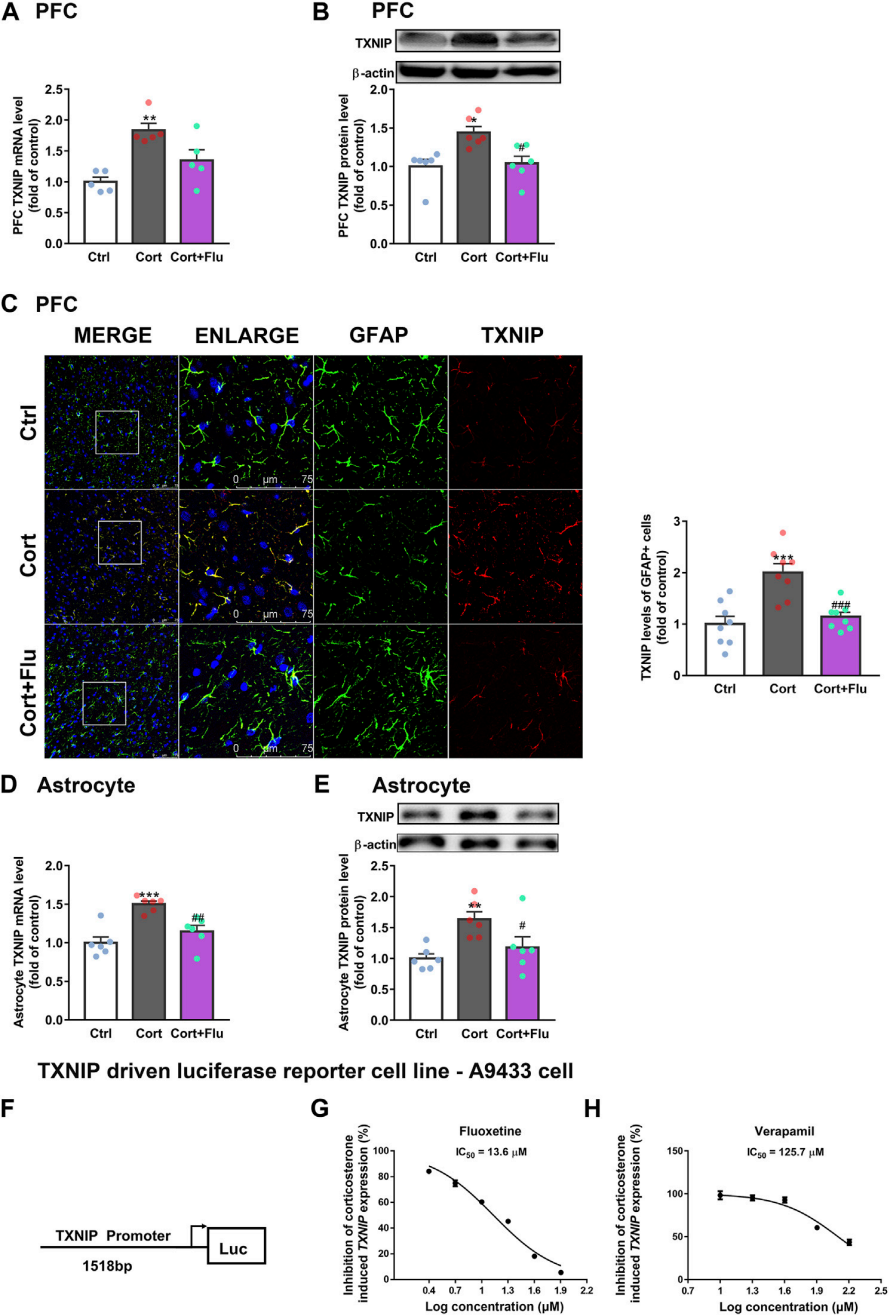
Fluoxetine restricts the transcription of *TXNIP* under corticosterone-induced depressive state. **(A,B)** TXNIP mRNA [**(A)**, *n* = 5 animals/group; *P* CortxCort + Flu = 0.2744)] and protein levels [**(B)**, *n* = 6 animals/group; *P* CortxCort + Flu = 0.0116)] in PFC of rats treated with fluoxetine (10 mg/kg). **(C)** Immunostaining of GFAP (green), TXNIP (red) and DAPI (blue) in PFC of rats treated with fluoxetine (10 mg/kg), scale bar = 75 μm. The fluorescence intensity was calculated by ImageJ, *n* = 8 fields/group. The fluorescence intensity of TXNIP (red) was normalized by GFAP (green); *P* CortxCort + Flu = 0.0006. **(D,E)**TXNIP mRNA [**(D)**, *n* = 6 cell cultures/group; *P* CortxCort + Flu = 0.0039)] and protein levels [**(E)**, *n* = 6 cell cultures/group; *P* CortxCort + Flu = 0.0466)] in fluoxetine (5 μM)-treated primary astrocytes. **(F)** A human *TXNIP* promoter (> 1518bp + 50 UTR) connecting with luciferase reporter was stably transfected into U87MG. **(G)** A9433 cells exposed to 100 nM corticosterone was treated with fluoxetine at different concentrations (2.5, 5, 10, 20, 40, and 80 μM, *n* = 3 cell cultures/group). **(H)** A9433 cells exposed to 100 nM corticosterone were treated with verapamil at different concentrations (10, 20, 40, 80, and 160 μM, *n* = 3 cell cultures/group). All data were expressed as mean ± SEM, **p < 0.01, ***p < 0.001 vs. Ctrl (control) group, ^#^ *p* < 0.05, ^##^ *p* < 0.01, ^###^ *p* < 0.001 vs. Cort (corticosterone) group (Normality of data was checked by Shapiro-Wilk test. Data with or without normal distribution were analyzed by one-way ANOVA or Kruskal-Wallis test followed by Dunnett’s post-hoc test for multiple comparisons).

Immunoprecipitation assay showed that TXNIP could interact with GLUT1 in normal primary astrocytes isolated from newborn rats (Supplementary Figure S3). Therefore, we analyzed the effect of fluoxetine on astrocytic TXNIP level *in vivo* and *in vitro*. Fluoxetine suppressed TXNIP mRNA (Figure 5A) and protein (Figure 5B) levels in PFC of depressive rats induced by repeated corticosterone injection. Furthermore, it reduced PFC astrocytic (GFAP+) TXNIP levels in this animal model (Figure 5C). Similarly, fluoxetine reduced TXNIP mRNA (Figure 5D) and protein (Figure 5E) levels in cultured primary astrocytes exposed to stress-level corticosterone.

As fluoxetine suppressed astrocytic TXNIP both in mRNA and protein levels, we speculated that fluoxetine reduced corticosterone-induced *TXNIP* transcription. To confirm this, we first developed a stable *TXNIP*-promoter-driven luciferase reporter cell line (U87MG), named A9433 cells. In A9433 cells, *TXNIP* promoter was connected with a luciferase reporter (Figure 5F). The inhibitory potential of fluoxetine on *TXNIP* transcription was assessed, and its significant decrease of luciferase activity was observed in corticosterone-exposed A9433 cells. Fluoxetine significantly inhibited corticosterone-induced *TXNIP* transcription in A9433 cells (IC_50_ = 13.6 μM) (Figure 5G). In this experiment, a reported *TXNIP* transcriptional inhibitor verapamil (Xu et al., 2012; Harper et al., 2019), presented IC_50_ value of 125.7 μM (Figure 5H).

**FIGURE 6:**
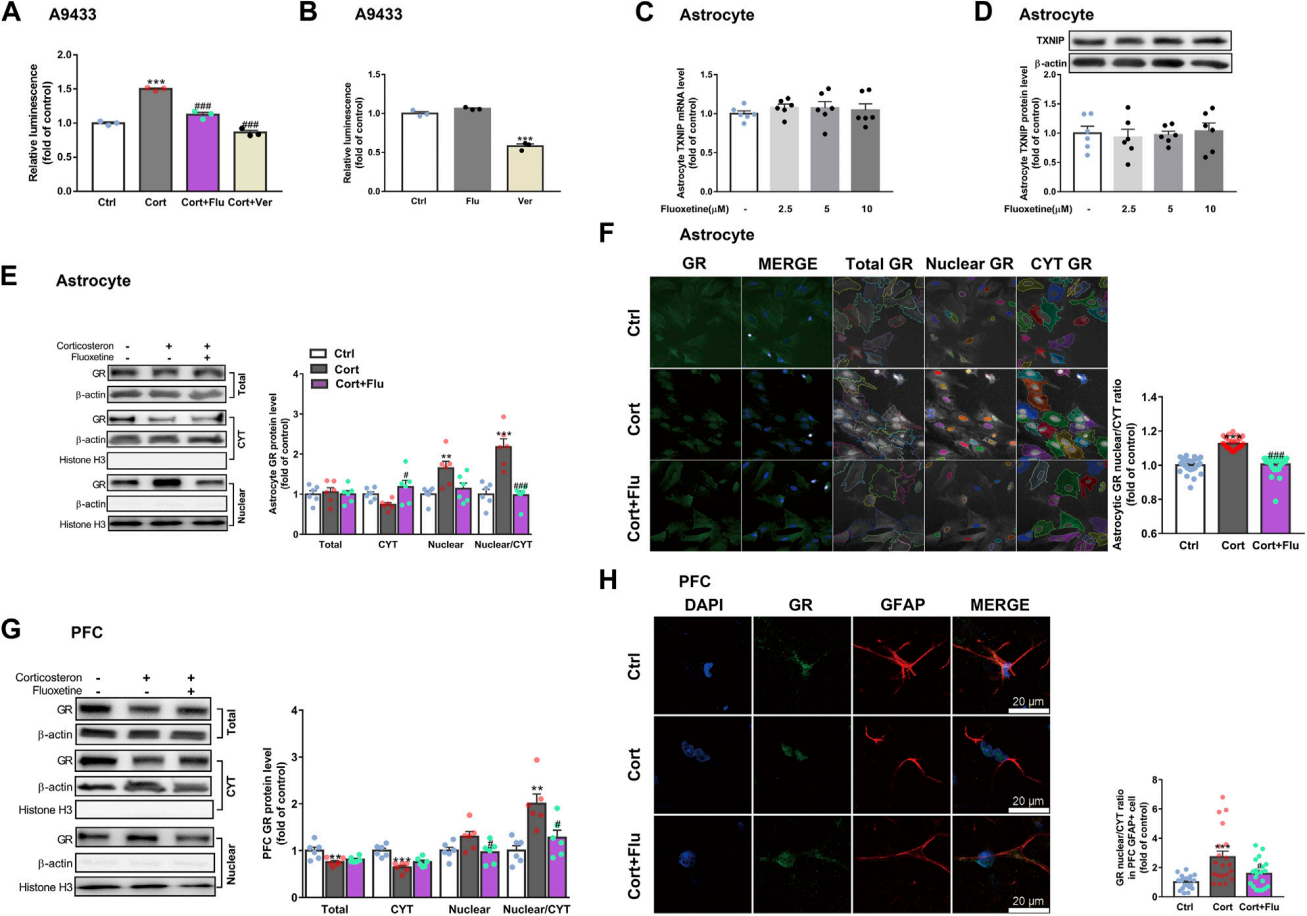
Fluoxetine restricts glucocorticoid receptor nuclear translocation of astrocytes *in vivo* and *in vitro.* **(A)** Fluoxetine (5 μM) and verapamil (80 μM) significantly reduced corticosterone-induced TXNIP expression in A9433 cells, *n* = 3 cell cultures/group; *P* CortxCort + Flu <0.0001; *P* CortxCort + Ver <0.0001. **(B)** Fluoxetine (5 μM) had no effect on TXNIP expression in normal A9433 cells, but verapamil (80 μM) significantly reduced TXNIP expression in normal A9433 cells, *n* = 3 cell cultures/group; *P* CtrlxFlu = 0.1597; *P* CtrlxVer < 0.0001. **(C)** TXNIP mRNA level of normal astrocytes without treatment or treated with fluoxetine (2.5, 5, and 10 μM), *n* = 6 cell cultures/group; *P* CtrlxFlu2.5 = 0.7240; *P* CtrlxFlu5 = 0.7796; *P* CtrlxFlu10 = 0.9324. **(D)** TXNIP protein level of normal astrocytes without treatment or treated with fluoxetine (2.5, 5, and 10 μM), *n* = 6 cell cultures/group; *P* CtrlxFlu2.5 = 0.9509; *P* CtrlxFlu5 = 0.9961; *P* CtrlxFlu10 = 0.9931. **(E)** GR (total, CYT and nuclear fractions) protein levels in corticosterone (100 nM) and fluoxetine (5 μM) treated primary astrocytes, *n* = 6 cell cultures/group; Total: *P* CortxCort + Flu = 0.9093; CYT: *P* CortxCort + Flu = 0.0163; Nuclear: *P* CortxCort + Flu = 0.0532; Nuclear/CYT: *P* CortxCort + Flu <0.0001. **(F)** Immunostaining of GR (green), and DAPI (blue) in corticosterone (100 nM) and fluoxetine (5 μM) treated primary astrocytes, *n* = 30 fields/group; *P* CortxCort + Flu <0.0001. **(G)** GR (total, CYT and nuclear fractions) protein levels in PFC of rats treated with corticosterone (40 mg/kg) and fluoxetine (10 mg/kg), *n* = 6 animals/group; Total: *P* CortxCort + Flu = 0.6424; CYT: *P* CortxCort + Flu = 0.1650; Nuclear: *P* CortxCort + Flu = 0.0484; Nuclear/CYT: *P* CortxCort + Flu = 0.0137. **(H)** Immunostaining of GR (green), GFAP (red) and DAPI (blue) in PFC of rats treated with corticosterone (40 mg/kg) and fluoxetine (10 mg/kg), scale bar = 20 μm, *n* = 20 cells/group; *P* CortxCort + Flu = 0.0390. All data were expressed as mean ± SEM, **p < 0.01, ***p < 0.001 vs. Ctrl (control) group, ^#^ *p* < 0.05, ^##^ *p* < 0.01, ^###^ *p* < 0.001 vs. Cort (corticosterone) group (Normality of data was checked by Shapiro-Wilk test. Data with or without normal distribution were analyzed by one-way ANOVA or Kruskal-Wallis test followed by Dunnett’s post-hoc test for multiple comparisons).

Of note, verapamil suppressed *TXNIP* transcription in A9433 cells with or without corticosterone stimulation, whereas, fluoxetine inhibited *TXNIP* transcription only in corticosterone stimulated-A9433 cells (Figures 6A,B). Fluoxetine showed no effect on TXNIP mRNA and protein levels of astrocytes isolated from newborn rats without corticosterone stimulation (Figures 6C,D). Thus, fluoxetine may stimulate PFC astrocytic glucose uptake and glycolysis in depressive rats *via* inhibiting corticosterone-induced *TXNIP* transcription.

### 3.4 Fluoxetine suppresses thioredoxin-interacting protein overexpression *via* restricting glucocorticoid receptor translocate into the nucleus

**FIGURE 7:**
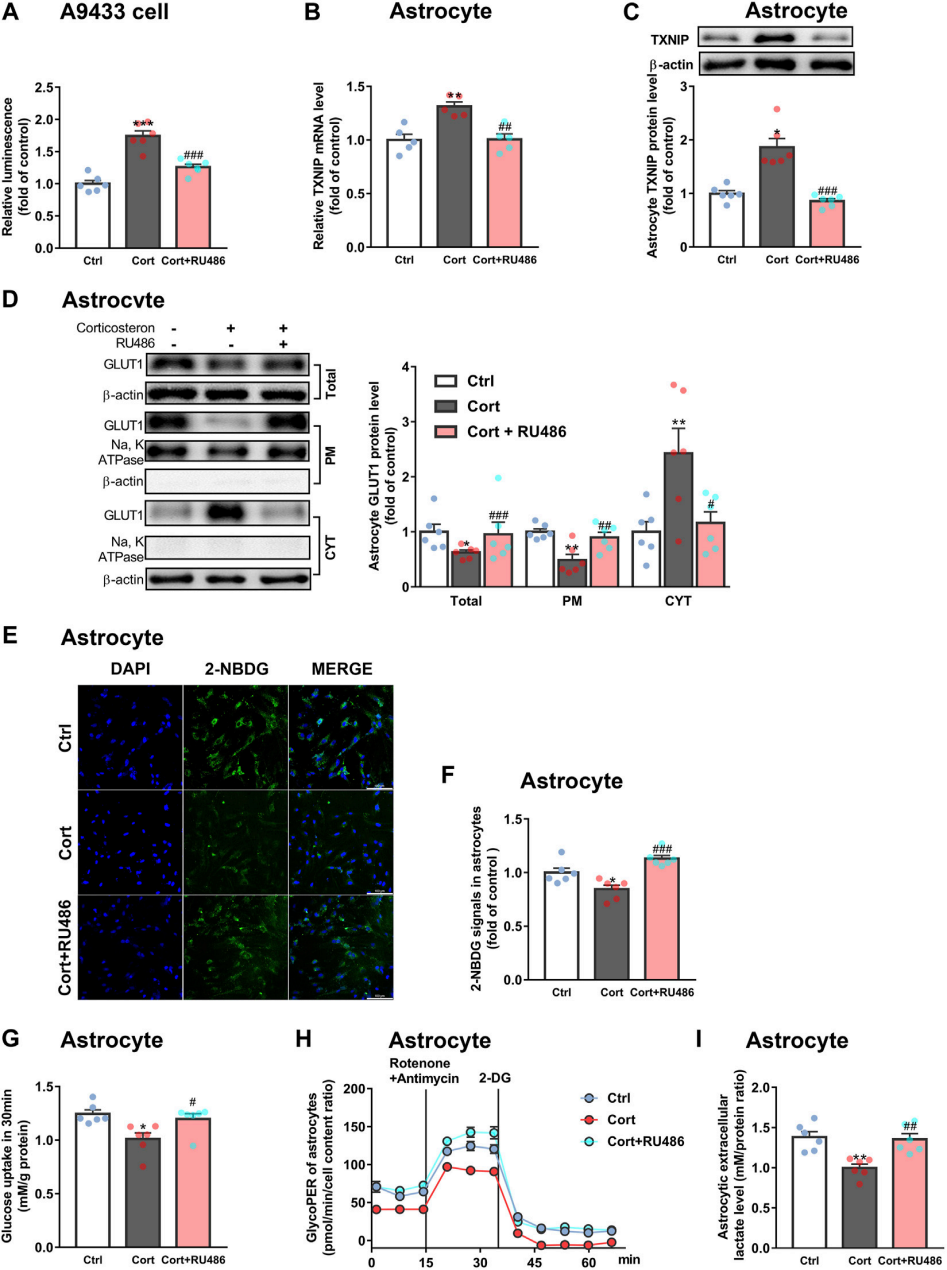
RU486 suppresses *TXNIP* transcription and improves astrocytic glucose uptake and glycolysis. **(A)** RU486 (1 μM) inhibited luciferase activity of A9433 cells obviously, *n* = 6 cell cultures/group; *P* CortxCort + RU486 < 0.0001. **(B,C)** RU486 (1 μM) reduced TXNIP mRNA [**(B)**, *n* = 5 cell cultures/group; *P* CortxCort + RU486 = 0.0017)] and protein [**(C)**, *n* = 6 cell cultures/group; *P* CortxCort + RU486 = 0.0007)] levels of primary astrocytes. **(D–I)** RU486 (1 μM) increased GLUT1 PM translocation [**(D)**, *n* = 6 cell cultures/group; Total: *P* CortxCort + RU486 = 0.0007; PM: *P* CortxCort + RU486 = 0.0094; CYT: *P* CortxCort + RU486 = 0.0188)], 2-NBDG uptake [**(E)**, scale bar = 100 μm; **(F)**, *n* = 6 cell cultures/group; *P* CortxCort + RU486 = 0.0001)], glucose uptake [**(G)**, *n* = 6 cell cultures/group; *P* CortxCort + RU486 = 0.0257)], glycolytic rate [**(H)**, *n* = 6 cell cultures/group)] and lactate release [**(I)**, *n* = 6 cell cultures/group; *P* CortxCort + RU486 = 0.0095)] of *in vitro* cultured primary astrocytes. All data were expressed as mean ± SEM, *p < 0.05, **p < 0. 01, ***p < 0.001 vs. Ctrl (control) group, ^#^ *p* < 0.05, ^##^ *p* < 0.01, ^###^ *p* < 0.001 vs. Cort (corticosterone) group (Normality of data was checked by Shapiro-Wilk test. Data with or without normal distribution were analyzed by one-way ANOVA or Kruskal-Wallis test followed by Dunnett’s post-hoc test for multiple comparisons).

Furthermore, we explored whether fluoxetine suppressed *TXNIP* transcription via GR. Fluoxetine was found to reduce GR nuclear translocation in corticosterone-exposed primary astrocytes *in vitro* (Figures 6E,F). Similarly, fluoxetine suppressed astrocytic (GFAP+) GR nuclear translocation in PFC of depressive rats induced by repeated corticosterone injection (Figures 6G,H). In fact, RU486 (a GR antagonist) inhibited corticosterone-induced *TXNIP* transcription in A9433 cells (Figure 7A), and TXNIP overexpression in primary astrocytes isolated from newborn rats (Figures 7B,C). Consistently, RU486 significantly promoted GLUT1 PM translocation (Figure 7D), 2-NBDG uptake (Figures 7E,F), glucose uptake (Figure 7G), glycolytic rate (Figure 7H) and extracellular lactate level in astrocytes exposed to corticosterone (Figure 7I). These data suggested that inhibition of GR could abolish the effect of corticosterone on *TXNIP* transcription and glucose metabolism of astrocytes. Thus, fluoxetine suppressed astrocytic TXNIP overexpression possibly *via* inhibiting GR nuclear translocation in corticosterone-induced depression.

## 4 Discussion

Clinically, the first-line antidepressant fluoxetine increases glucose metabolism rate of right superior frontal gyrus in patients with first-episode depression (Zhang et al., 2006). Its 12 week-treatment improves PFC glucose metabolism of impulsive aggression patients (New et al., 2004). Here, we found that fluoxetine improved PFC glucose metabolism, in parallel with its antidepressant behavioral effect in corticosterone-stimulated rats.

Fluoxetine could increase glucose uptake of peripheral blood mononuclear cells (Stapel et al., 2019) and improve peripheral glucose metabolism in streptozotocin-induced diabetes of rats (Yang et al., 2020). Our previous study showed that fluoxetine alleviated glucose intolerance in chronic unpredictable mild stress-induced animal model of depression (Pan et al., 2013). But the mechanism by which fluoxetine improves glucose metabolism in depression is unclear. The “endfeet” process of astrocytes covers cerebral blood vessels and takes up glucose from blood vessels (MacVicar and Newman, 2015; Nortley and Attwell, 2017). In this study, fluoxetine mainly increased 2-NBDG signals of SR101 + astrocytes in *ex vivo* cultured PFC slices from corticosterone-induced depressive rats. Consistently, fluoxetine significantly increased glucose metabolism in corticosterone-exposed astrocytes *in vitro*. These observations indicated that fluoxetine mainly enhanced PFC astrocytic glucose metabolism in corticosterone-induced depression. In astrocytes, low activity of pyruvate dehydrogenase complex restricts pyruvate to enter tricarboxylic acid cycle (Halim et al., 2010). Besides, high fructose-2,6-bisphosphatase-3 and pyruvate kinase muscle isoform-2 levels guarantee high glycolytic rate of astrocytes (Bolanos, 2016). Lactate, the end-product of astrocytic glycolysis, can be transferred to neurons (Magistretti and Allaman, 2018) to support synaptic plasticity (Murphy-Royal et al., 2020). In our study, fluoxetine treatment increased PFC astrocytic lactate release *in vivo* and *in vitro*, modulating astrocytic glycolysis in corticosterone-induced depression, which may be one possible reason that fluoxetine could improve neuronal plasticity (Zavvari et al., 2020).

Then, we explored how fluoxetine regulated astrocytic glycolysis in corticosterone-induced depressive state. In our study, fluoxetine directly increased glucose uptake and glycolysis in *in vitro* corticosterone-exposed primary astrocytes, suggesting that fluoxetine could increase astrocytic glucose metabolism independently of serotonin in corticosterone-induced depressive state. Fluoxetine is reported to reduce hippocampal TXNIP protein levels in rats exposed to chronic unpredictable mild stress (Song et al., 2018). In this study, we firstly detected the influence of fluoxetine on astrocytic TXNIP level in depression, and observed that fluoxetine increased TXNIP-GLUT1 pathway-mediated glucose uptake in corticosterone-stimulated PFC astrocytes *in vivo* and *in vitro*. More importantly, compared with the reported *TXNIP* transcriptional inhibitor verapamil, fluoxetine showed better inhibitory activity on *TXNIP* transcription in corticosterone-exposed A9433 cells.

Dysfunctional glucocorticoid signaling is thought to contribute to some mood disorders such as major depression (McEwen, 2005). Glucocorticoid can bind to glucocorticoid receptor (GR) or mineralocorticoid receptor (MR) to act as transcriptional regulators. Although presenting higher affinity for glucocorticoid than GR (Reul et al., 2015), MR is largely occupied under normal glucocorticoid condition, whereas high glucocorticoid, such as levels-associated stressful experiences, mainly impacts GR-mediated glucocorticoid signaling (de Kloet et al., 2018). Therefore, more studies focus on GR function in the treatment of depression (Camargo et al., 2020; Li et al., 2020). In addition, astrocytes are found to exhibit lower levels of MR than neurons, thus, *in vitro* astrocytes cultured with glucocorticoid (dexamethasone) may present much more transcriptional changes than mineralocorticoid (aldosterone) (Piechota et al., 2017). Functional glucocorticoid response elements are presented in murine *TXNIP* promoter (Wang et al., 2006). Glucocorticoids combined with GR, translocate into nucleus, then bind to glucocorticoid response elements, and induce the transcription of *TXNIP* in human and murine cell line (Wang et al., 2006; Harada et al., 2015). Fluoxetine is reported to restrict the translocation of GR from cytoplasm to nucleus in hippocampus of corticosterone-treated mice (Zhang et al., 2016). Hippocampus and frontal cortex TXNIP protein levels are obviously increased in mice exposed to chronic unpredictable stress (Zhou et al., 2019). However, few study has explored the relationship between GR and TXNIP in depressive condition. In our study, fluoxetine obviously inhibited *TXNIP* transcription in primary astrocytes and A9433 cells with corticosterone exposure. Of note, fluoxetine showed no effect on TXNIP mRNA and protein levels in primary astrocytes without corticosterone stimulation, indicating that fluoxetine-affected *TXNIP* transcription may be related with glucocorticoid-GR. More importantly, we found that GR antagonist RU486 significantly suppressed *TXNIP* transcription in A9433 cells and primary astrocytes with corticosterone exposure. It obviously increased glucose uptake and glycolysis in corticosterone-exposed primary astrocytes. We also found that fluoxetine reduced GR nuclear translocation of astrocytes in PFC of repeated corticosterone injection-induced depressive rats. Fluoxetine restricted GR nuclear translocation in corticosterone-stimulated primary astrocytes *in vitro*. These results suggested that fluoxetine might inhibit *TXNIP* transcription in corticosterone-exposed astrocytes through modulation of GR translocation. Although cortisol/corticosterone is proved as a risk factor for depression (Zajkowska et al., 2022), the effect of fluoxetine on GR translocation in other animal model of depression is needed to elucidate in future.

Taken together, our research firstly explored the effect of fluoxetine on astrocytic GR level and location in corticosterone-induced depressive state, and suggested that fluoxetine modulated GR-TXNIP-GLUT1 pathway to improve astrocytic glucose uptake and glycolysis in corticosterone-induced depression.

## 5 Conclusion

**FIGURE 8:**
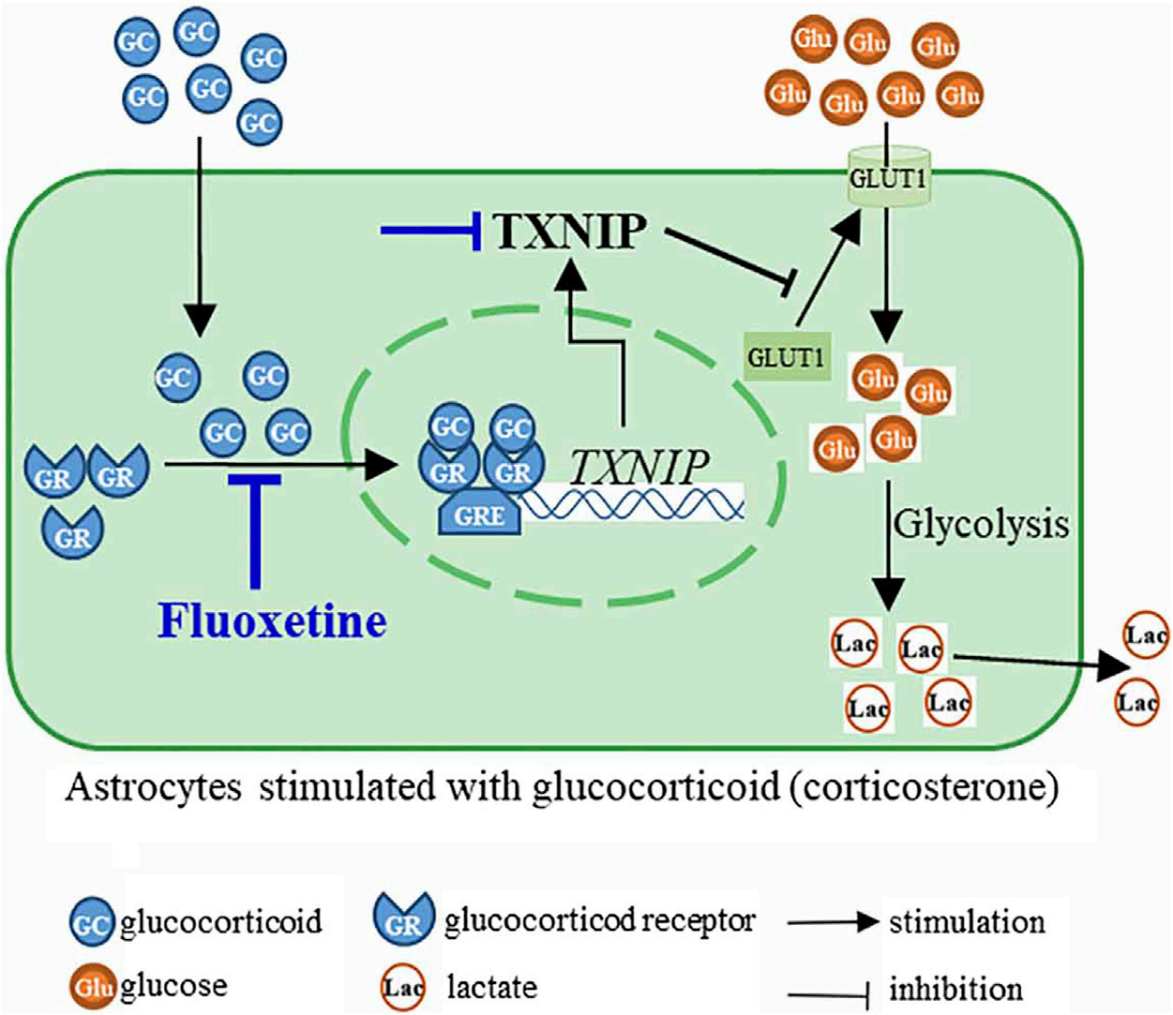
Fluoxetine inhibited nuclear translocation of glucocorticoid receptor (GR) to suppress the transcription of *TXNIP*, and then modulated TXNIP-GLUT1 pathway, resulting in the improvement of astrocyte glucose uptake and glycolysis in corticosterone-induced depression.

This study finds that fluoxetine recovers PFC astrocytic glucose uptake and glycolysis in corticosterone-induced depression through GR-TXNIP-GLUT1 pathway. Modulation of astrocytic glucose metabolism by fluoxetine may be suggested as a novel antidepressant strategy (Figure 8).

## Data Availability

Raw data in this manuscript can be obtained by contacting with corresponding author.
