# Supplementary material for: Fluoxetine increases astrocytic glucose uptake and glycolysis in corticosterone-induced depression through restricting GR-TXNIP-GLUT1 Pathway
Source: Front Pharmacol. 2022 Aug 29;13:872375. doi: 10.3389/fphar.2022.872375 (PMC9465171; doi:10.3389/fphar.2022.872375)
Supplement: Supplementary file 1 [file DataSheet1.docx]

Fluoxetine Increases Astrocytic Glucose Uptake and Glycolysis in Corticosterone-Induced Depression Through Restricting GR-TXNIP-GLUT1 Pathway

Shu-Man Pan^1^· Yi-Fan Zhou^1^ ·Na Zuo^1^· Rui-Qing Jiao^1^· Ling-Dong Kong^1, 2*^· Ying Pan^1, 2*^

1. State Key Laboratory of Pharmaceutical Biotechnology, School of Life Sciences, Nanjing University, Nanjing 210023, Jiangsu Province, P. R. China
2. State Key Laboratory of Natural Medicines, China Pharmaceutical University, Nanjing 210009, Jiangsu Province, P. R. China

🖂 Ying Pan

School of Life Sciences

Nanjing University

Nanjing, 210023

P. R. China

Tel. 86-25-89681373

Email: pany@nju.edu.cn

🖂 Ling-Dong Kong

School of Life Sciences

Nanjing University

Nanjing, 210023

P. R. China

Tel. 86-25-83594691

Email: kongld@nju.edu.cn

Supplementary materials and methods

**Cell viability assay**

Primary astrocytes were seeded in 96-well cell culture plate. When cell confluence reach about 60-70%, corticosterone (100 nM) or fluoxetine (2.5, 5, 10, 20, 40, 80, 160μM) were added into culture medium. 72 h after corticosterone and fluoxetine treatment, WST-8 reagent was added into cell culture plate according to manufactures’ instruction (C0038, Beyotime). Then, cell culture plate was incubated in 37 ℃ for 2 h. OD_450_ and OD_650_ were measured by microplate reader. Cell viability of primary astrocytes was calculated according to OD_450_ and OD_650_ value.

**Immunoprecipitation assay**

Primary astrocytes were seeded in T75 cell culture flasks. When cell confluence reach about 60-70%, corticosterone (100 nM) and fluoxetine (5 μM) were added into culture medium. 72 h after corticosterone and fluoxetine treatment, primary astrocytes were harvested and lysed by ultrasound. Then, the procedures of immunoprecipitation assay were conducted following the manufacture’s instruction of commercial kit (PK10007, Proteintech). Briefly, 4 μg TXNIP antibody (#14715, CST) was added into 2 mg lysate sample in spin column. The same amount IgG was used as negative control. The spin columns were rotated gently at 4 ^o^C overnight to allow the formation of antigen-antibody complexes. Then, 50 μL Protein A sepharose beads slurry were added into spin columns. After washing and elution, samples were collected and detected by Western blot assay.

Table S1. The sequence of human *TXNIP* promoter

| **The sequence of human *TXNIP* promoter (＞1518bp + 50 UTR)** |
| --- |
| ATTATCTTCCTCTTTCCAGCACAAGGAATTAAATAATAATCTTCTGATACCCCAGCACAGATATAGGAAGGGTCTATGAATCAAATAAAAGGAGGAAGAATCCCTGCACTTTTGCATACAGGTTTTTCCTATTACATTTAAGAGTAAGTTCTCAGTACTCAGCTCCTAAAGCATCTCACAGCCAGCAGGGGAAAATCCATCTGACAGCTGGCCAAACGAAACCAACAAAGAATGAAGAGAGAGGGTAGGGTCTCTTCTGGCTTGAATTTATAGTGCTCTGTTGACCGATCTTTCTTCTCTTTTCCTTCTTACTGTTTTCTAAACCGTTTAGGGAAAAACCTTTGAAAATAGTTTTTAAAATTGTTCCTTCAAACAGGTGTGTGGCCATCTCTCCACTGAAAATTTGGATATAAACAAGAGGACTTTCTCACTTTTAACCAGATTTATGGATGTACATTTGATTTAGTGAGTTGGAAGAGGGGATGGAGACAAGAAAGAGTGAACATAACTGGAAAAAAGTGAAAAGAGTGAAGCATCCTTTTTTTCCCGCTTTTCCTTTTCCTCCAGAAGCAGGTAGGAAGTGGGAGATAATGAGCGCCTGGACACACCTCACTAAAGAGGTAATGAGGTAAATGGGGAACACAGACAAAGTGTTCCCCAACTTTGCAGGTAGAAATTGAAGAGATGACAGGATAAGCAACAGGATGTAAACACGCCCCTCCTATTTCCGTTCCACAGAACAGAGAGAACAGAAGAGAGGGTACAAGCTGGGGGTGGGTGACGAACAGCACAGGCACGCAGCCCCCAGCCCTAGCCCCAAGGGATTGGAACGGGAAGGAGAAGACATCGGTCCTACACACACAATGAGGCCTGAAAGTTCTCCTTTCCCTCAGAGACGGTGGTGTTTTTTATACTTAATAGGGATGCGGGGCAAGAGAAGGACAAAGGGCTGTTCCTGAACAGTAACACCAAGCATTCTGCGCTCCACAGCCCCAAACCTGAAAGTATTCTTGGAGCTATGGGATTTTCACACACTTGCTATTTATGAGCCAGGAATAACGACAGGCTCTAAAGTAACTGCACTGGCTAAGACTAGGCATGAAATTCCCTCATAAGCACATTTTCCTTTTACCTCAAAACACCGCTCTCAGACCAGAAACGTCCACACCCGCCCTCCGATGGCCTGTCGCCCTGGCTAGGTTTTAGGGTCAGTGGGATCCTCCTTCCACTGGACCCGGGAGAAGACGCTCAACAGCCCCCTCCTTCCCCTCCTTCCTCTCCTTCCTCTCCTTCCCCCCTCCCTGCGCCGCTCCAGAGCGCAACAACCATTTTCCCAGCCAGGAGCACACCGTGTCCACGCGCCACAGCGATCTCACTGATTGGTCGGGCTCCTGGTAAACAAGGACCGGGCAGCCAATGGGAGGGATGTGCACGAGGGCAGCACGAGCCTCCGGGCCAGCGCTCGCGTGGCTCTTCTGGCCCGGGCTACTATATAGAGACGTTTCCGCCTCCTGCTTG |

Table S2. Brian ^18^F-FDG PET standard uptake values

| Group/brain regions | Amygdala_l | Striatum_l | FAC_l | mPFC_l | Hippo_AD_l | Hippo_P_l | Hypothalamus_l |
| --- | --- | --- | --- | --- | --- | --- | --- |
| Ctrl | 2.12 ± 0.08 | 2.72 ± 0.04 | 2.19 ± 0.42 | 2.75± 0.55 | 2.38 ± 0.11 | 2.48 ± 0.22 | 2.53 ± 0.05 |
| Cort | 2.04 ± 0.25 | 2.68 ± 0.26 | 1.77 ± 0.20 | 2.20 ± 0.22 | 2.27 ± 0.19 | 2.26 ± 0.23 | 2.48 ± 0.22 |
| Cort + Flu | 2.17 ± 0.13 | 2.80 ± 0.12 | 2.11 ± 0.34 | 2.84 ± 0.64# | 2.61 ± 0.35 | 2.66 ± 0.40 | 2.72 ± 0.25 |
| Group/brain regions | Amygdala_r | Striatum_r | FAC_r | mPFC_r | Hippo_AD_r | Hippo_P_r | Hypothalamus_r |
| Ctrl | 2.28 ± 0.11 | 2.68 ± 0.01 | 2.16 ± 0.47 | 2.79 ± 0.61 | 2.45 ± 0.19 | 2.60 ± 0.42 | 2.60 ± 0.18 |
| Cort | 2.17 ± 0.19 | 2.68 ± 0.23 | 1.68 ± 0.20 | 2.18 ± 0.20* | 2.26 ± 0.20 | 2.18 ± 0.21 | 2.42 ± 0.27 |
| Cort + Flu | 2.30 ± 0.13 | 2.77 ± 0.09 | 2.12 ± 0.44 | 2.91 ± 0.73# | 2.62 ± 0.36 | 2.65 ± 0.47 | 2.68 ± 0.26 |
| Values are mean ± SEM (n = 4).  ^*^*P* < 0.05 *vs.* Ctrl (control) group; ^#^*P* < 0.05 *vs.* corticosterone-stimulated group. | | | | | | | |


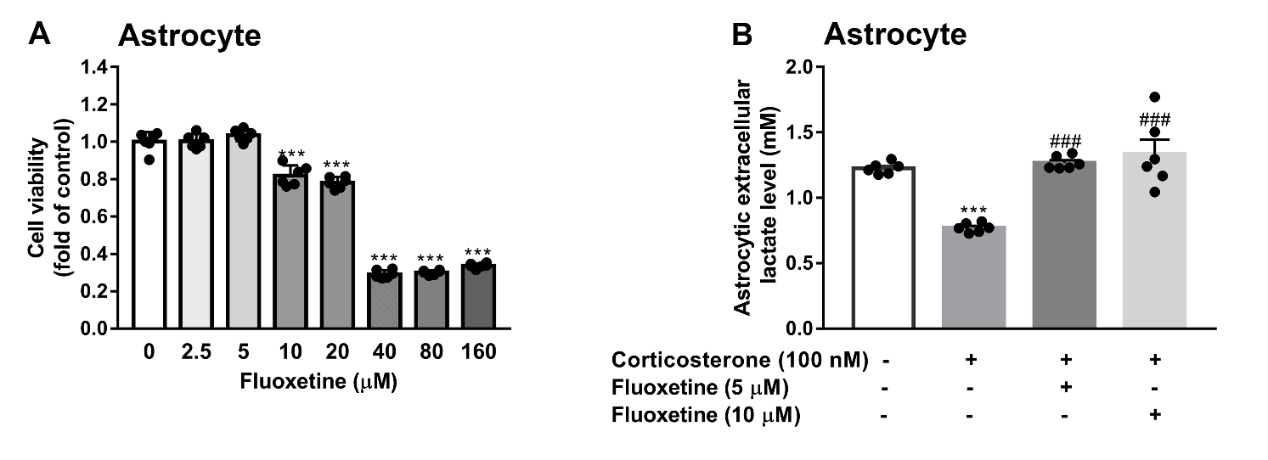


**FIGURE S1** The effect of fluoxetine on astrocytic cell activity and lactate release. The effect of fluoxetine (2.5, 5, 10, 20, 40, 80 and 160 μM) on cell activity of astrocytes isolated from newborn rats, n = 6 cell cultures/group **(A)**. Fluoxetine (5 and 10 μM) increased supernatant lactate level of astrocytes isolated from newborn rats, n = 6 cell cultures/group **(B)**. **Fluoxetine (5 μM) improved astrocytic glycolysis and had no obvious effect on cell activity. Therefore, we choose 5 μM as the dosage in following experiments.** All data were expressed as mean ± SEM. ^***^*P* < 0.001 vs. Ctrl (control) group, ^###^*P* < 0.001 vs. Cort (corticosterone) group (One-way ANOVA followed by Dunnett’s post-hoc test).


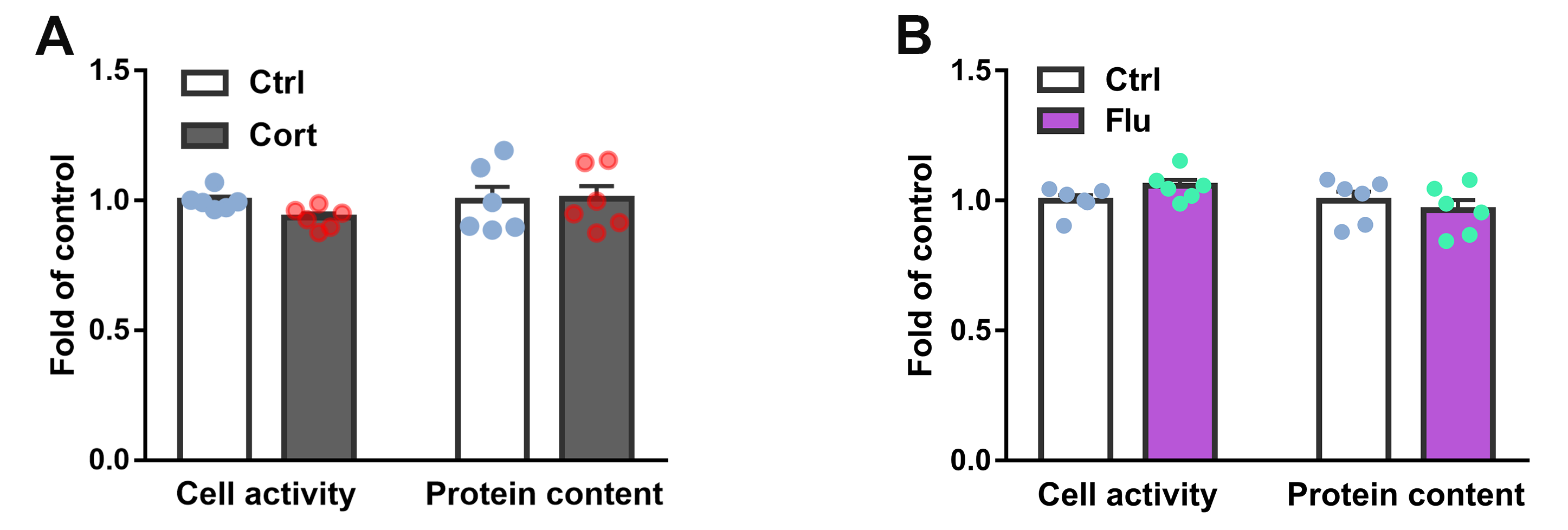


**FIGURE S2** 100 nM corticosterone or 5 μM fluoxetine has no obvious effect on cell activity and protein content of astrocytes. After stimulation of corticosterone (100 nM), the cell activity or protein content in astrocytes isolated from newborn rats was detected by CCK-8 or BCA protein assay kit, n = 6 **(A)**. After exposure to fluoxetine (5 μM), the cell activity or protein content in astrocytes isolated from newborn rats was detected by CCK-8 or BCA protein assay kit, n = 6 **(B)**. All data were expressed as mean ± SEM.


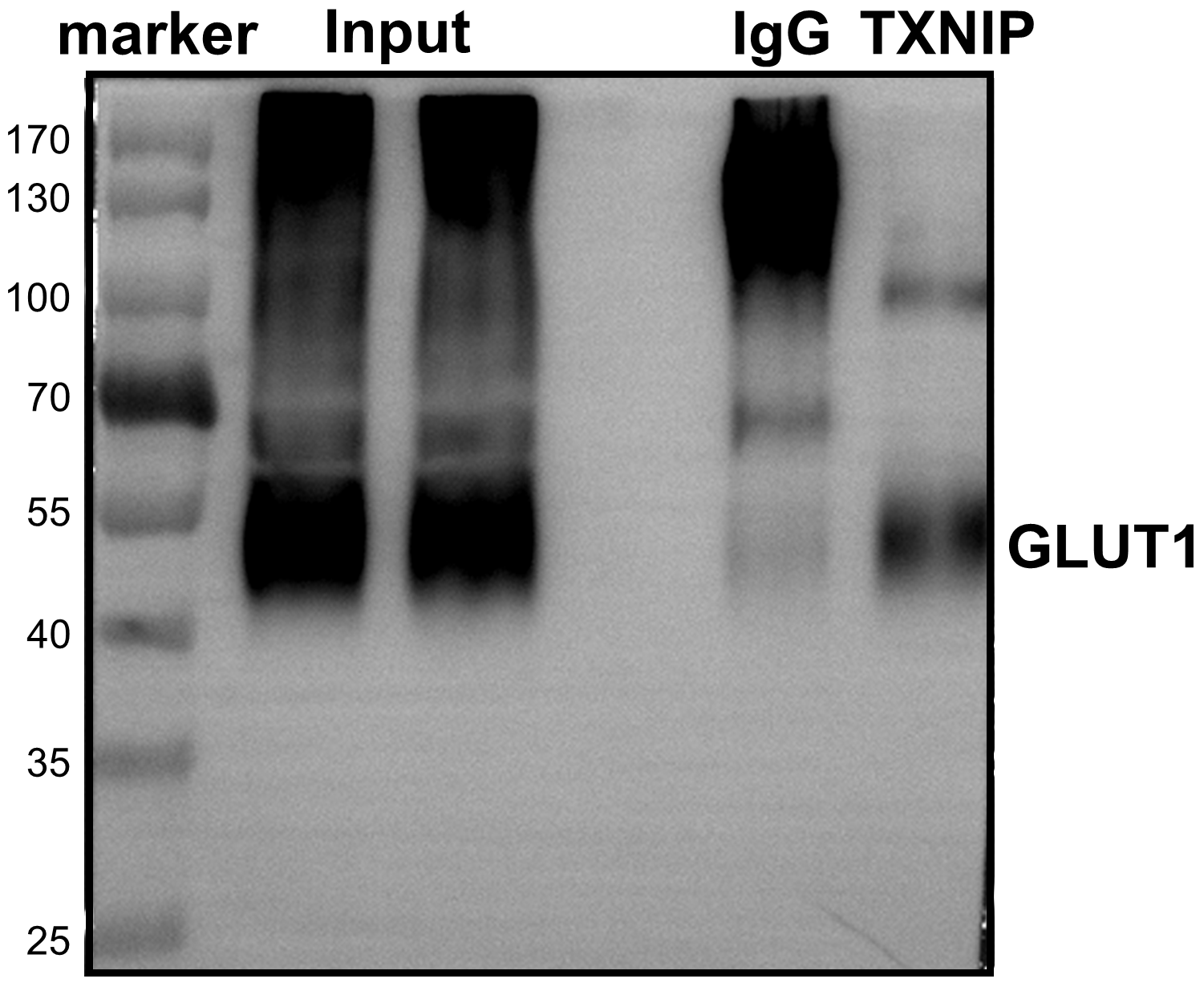


**FIGURE S3** Immunoprecipitation assay of TXNIP and GLUT1 in primary astrocytes isolated from PFC of newborn rats.
